# Prevalence of Nonalcoholic Steatohepatitis and Associated Fibrosis Stages Among US Adults Using Imaging-Based vs Biomarker-Based Noninvasive Tests

**DOI:** 10.36469/001c.92223

**Published:** 2024-02-14

**Authors:** Jesse Fishman, Tom O’Connell, Christina M. Parrinello, Jonathan J. Woolley, Eric Bercaw, Michael R. Charlton

**Affiliations:** 1 Madrigal Pharmaceuticals, West Conshohocken, Pennsylvania, USA; 2 Medicus Economics, Boston, Massachusetts, USA; 3 Pine Mountain Consulting, LLC, Redding, Connecticut, USA; 4 Center for Liver Diseases, The University of Chicago Medicine, Chicago, Illinois, USA

**Keywords:** nonalcoholic fatty liver disease, nonalcoholic steatohepatitis, noninvasive tests, prevalence, United States

## Abstract

**Introduction:** Nonalcoholic fatty liver disease (NAFLD) is believed to be the most common chronic liver disease worldwide. Therapies are under development for nonalcoholic steatohepatitis (NASH), the progressive form of NAFLD, such that the prevalence of NASH with liver fibrosis, which is likely to require treatment, may be of interest to healthcare decision makers. Noninvasive tests are used in initial screening for NASH, as well as in observational studies of NASH prevalence. However, existing evidence does not address how estimated prevalence varies with different noninvasive tests. This analysis estimated the prevalence of NASH among US adults and assessed variation with different noninvasive tests.

**Methods:** A cross-sectional analysis was conducted using the 2017–March 2020 National Health and Nutrition Examination Survey cycle. Participants with presumed NAFLD (steatosis and without alternative causes of liver disease) were identified, among whom NASH was predicted based on FAST score, Fibrosis-4 (FIB-4), and AST-to-Platelet Ratio Index (APRI) cutoffs across 11 scenarios. Among NASH participants, fibrosis stages were explored based on distribution across the spectrum of liver-stiffness measurements.

**Results:** Among participants with complete data for the analysis (N=6969), prevalence of presumed NAFLD was 25.6%. Within presumed NAFLD, prediction of NASH using imaging-based NIT cutoffs yielded estimated prevalence of 1.3%-4.8% (3.3 million-12.2 million) based on FAST score cutoffs from 0.35-0.67. Using biomarker-based NIT cutoffs yielded estimated prevalence of 0.4%-12.3% (1.0 million-14.5 million) based on FIB-4 cutoffs from 0.90-2.67, and 0.1%-1.9% (0.2-5.0 million) based on APRI cutoffs from 0.50-1.50.

**Conclusion:** Prevalence of NASH among US adults was estimated to range from 1.3% to 4.8% when predicted using imaging-based noninvasive test values for participants with presumed NAFLD, generally aligning with estimates in the literature of prevalence of biopsy-confirmed NASH. Use of biomarker-based noninvasive test values for prediction of NASH yielded a wider range of estimates with FIB-4, and a considerably lower range of estimates with APRI.

## INTRODUCTION

Nonalcoholic fatty liver disease (NAFLD) refers to a condition in which excess fat is stored in the liver (hepatic steatosis) and is not attributable to other causes of chronic liver disease.^1,2^ The prognosis of NAFLD is heterogeneous, with only a subset of the prevalent population likely to progress to the most serious clinical outcomes. NAFLD is classified into nonalcoholic fatty liver (NAFL) and nonalcoholic steatohepatitis (NASH), distinguished from NAFL by presence of inflammation and hepatic injury^1,2^ and, in some instances, progressive fibrosis, which may lead to cirrhosis and attendant complications, including decompensation (ie, liver failure) and hepatocellular carcinoma.^3^ Several studies suggest that among the histological features of NAFLD/NASH, fibrosis stage is the most important prognostic factor for predicting liver-related morbidity and mortality.^4–6^

Despite the condition’s clinical significance, there are currently no regulatory-approved therapies for NASH.^7,8^ However, several agents are in development, with 5 phase 3 studies expected to complete collection of histological endpoints before the end of 2024 (aramchol, resmetirom, obeticholic acid, belapectin, lanifibranor, efruxifermin), and completion of other clinical outcome studies for obeticholic acid in 2025 and for semaglutide in 2028.^8^ With the emergence of therapeutic interventions, identification of NASH with active steatohepatitis and moderate-to-significant fibrosis, suggesting elevated risk of progression to cirrhosis, is expected to inform treatment eligibility.^8^ The increasing focus on accurate and scalable screening processes to identify treatment-eligible NASH is reflected in the growing evidence base addressing the use of noninvasive tests for prediction of “at-risk” NASH, defined as NAFLD Activity Score (NAS) (ranging from 0-8, as the sum of scores for degree of steatosis, ballooning of liver cells, and lobular inflammation^9^) ≥4 and fibrosis stage ≥2.^8^

As NAFLD is believed to be the most common chronic liver disease worldwide,^6^ the prevalence of treatment-eligible patients (ie, those with NASH with significant fibrosis) may be of particular interest to healthcare decision makers to inform assessment of the size of eligible patient populations and the financial and healthcare resource utilization impacts of screening. Meta-analysis of prevalence studies has previously estimated the global prevalence of NAFLD at 25% (95% confidence interval [CI]: 22%-29%). Estimates of the prevalence of NASH vary significantly with several factors, including the modalities used for case identification and the populations in which prevalence is assessed. For example, while some studies suggest that NASH is present in approximately one-third of NAFLD patients,^10,11^ the aforementioned meta-analysis estimated significantly higher (59%; 95% CI: 48%-70%), which the authors attributed to selection bias,^6^ as studies included in the meta-analysis required diagnosis of NASH via liver biopsy, which is only performed on suspicion of progressive disease. When considering prevalence estimates based on voluntary biopsy, the authors of the meta-analysis estimated that NASH may be present in 7%-30% of patients with NAFLD, implying general population prevalence of 1.5%-6.5%. However, a recent prospective study found prevalence of NASH of 14% (95% CI: 12%-17%) upon voluntary biopsy among Americans over the age of 45 attending a military medical center in Texas for colonoscopy,^12^ illustrating the potential range of estimates of the prevalence of NASH in the general population.

While liver biopsy is required for definitive diagnosis of NASH, due to the intrinsic invasive nature, cost, and risk of biopsy, noninvasive tests (NITs) are recommended in initial steps for screening of NAFLD/NASH with elevated risk of progression, including tests of blood-based biomarkers and imaging tests. Common blood-based biomarkers include individual markers such as aminotransferase levels, platelet count, coagulation parameters, gamma-glutamyl transferase, total bilirubin, α^2^-macroglobulin, and α^2^-globulin (haptoglobin).^13^ Individual biomarkers have also been combined into panels,^13^ and considered along with demographic and clinical data to create algorithms for staging and risk prediction, such as the commonly-used Fibrosis-4 (FIB-4) index and AST-to-Platelet Ratio Index (APRI).^13,14^ More recently, imaging-based NITs have gained adoption,^7,15,16^ such as those based on vibration-controlled transient elastography (VCTE) conducted with FibroScan.^7^

For screening for NAFLD/NASH with elevated risk of progression, recent clinical guidance statements have recommended tiered use of FIB-4 and VCTE, including the European Association for the Study of the Liver,^17^ the American Association of Clinical Endocrinology (AACE),^18^ and the American Association for the Study of Liver Diseases (AASLD).^19^ Aligning with this approach, efforts have recently been made to combine blood-based biomarkers with imaging-based NITs to improve the accuracy of staging risk of NAFLD progression.^7,13^ In particular, the FibroScan + AST (FAST) score was developed to predict at-risk NASH, based on liver stiffness measurement (LSM) by VCTE as an imaging measure of fibrosis, controlled attenuated parameter (CAP) by VCTE as an imaging measure of steatosis, and AST as a biomarker of activity.^20^ The FAST score was reported to have satisfactory predictive performance vs biopsy-based evaluation among patients at secondary/tertiary care centers included in its derivation population in England (C-statistic 0.80; 95% CI: 0.76–0.85), subsequently in 7 international external validation populations (C-statistic range 0.74–0.95),^20^ and in a recent systematic review and meta-analysis of studies using the FAST score for identification of fibrotic NASH.^21^ In the US, among patients with biopsy-proven NAFLD in the multicenter NASH Clinical Research Network Adult Database 2 cohort study, the FAST score has been reported to have higher predictive accuracy for at-risk NASH than other NITs (C-statistic 0.81 vs 0.74 for APRI, 0.73 for FIB-4, and 0.67 for the NAFLD Fibrosis Score [NFS]).^22^ However, limited positive predictive value of NITs (ie, resulting in false-positive predictions) has been documented when applied in the general population vs cohorts with evidence of NAFLD or clinical suspicion for NASH.^8,23^

Considering the use of NITs in initial steps of screening for NASH with significant fibrosis, analysis of their prevalence may inform upper-bound estimates of the size of eligible patient populations for emerging therapeutic interventions. For example, a recent study estimated prevalence of NAS ≥4 and fibrosis stage ≥2 in the US adult population to range from 1.2%-5.8%, based on application of the FAST score rule-out (0.35) and rule-in (0.67) cutoffs to data from the National Health and Nutrition Examination Survey (NHANES) 2017-2018 cycle.^24^ However, existing evidence does not address how the estimated prevalence of NASH varies with the use of different NITs that have historically been considered for screening, nor the associated variation in estimated prevalence across fibrosis stages, which may affect eligibility for interventions. Different NITs have been used for estimation of prevalence across different studies/populations,^24–30^ but evidence is limited on the impact of different NITs in a single, consistent population. In this analysis, we therefore sought to estimate the prevalence of NASH using several NITs commonly used for screening,^22^ including the FIB-4 and APRI biomarker-based NITs, and the FAST score imaging-based NIT, utilizing nationally representative data for the US from the most recent NHANES 2017–March 2020 survey cycle. Variation in estimated fibrosis stages based on the NIT used was also explored.

## METHODS

### Study Design and Data

A cross-sectional analysis was conducted of the 2017–March 2020 pre-pandemic continuous NHANES survey cycle^31^ to estimate the prevalence of NASH and the distribution of prevalence across fibrosis stages. This report of the analysis was developed in accordance with the CROSS checklist for survey research (see **Supplementary Material**).^32^

The NHANES, a population-based survey conducted by the National Center for Health Statistics (NCHS), collects information on the health and nutritional status of the civilian, non-institutionalized (eg, not in prison, nursing home) population in the US.^33^ Since 1999, data have been continuously collected and released in 2-year cycles, including blood-based biomarkers used as NITs for staging and identification of NASH. Due to the COVID-19 pandemic, the 2019-2020 survey cycle was not completed and the data collected were not nationally representative. However, the NCHS combined the 2019-2020 partial data with the previous 2017-2018 survey cycle data to create the 2017-March 2020 pre-pandemic cycle. This survey cycle covers a 3.2-year period (compared with the previous 2-year survey cycles) and can be weighted to provide nationally representative estimates similar to previous survey cycles.^31^ The 2017-2020 survey cycle includes liver ultrasound transient elastography data containing measures of liver stiffness and steatosis from VCTE. The continuous NHANES protocols were approved by the NCHS Institutional Review Board. Informed consent was obtained from all participants.

### Analytic Framework

The population for this analysis included adult participants in NHANES who completed the medical examination and were not pregnant, as pregnancy may affect biomarkers used to identify conditions in the analysis (eg, aminotransferase levels^34^). A complete-case approach was used for the analysis (ie, any participant with a missing value for an analytic variable required in the base-case or scenario analyses was excluded). Accordingly, participants included in the analysis were those with complete data for determination of causes of liver disease other than NAFLD (ie, excessive alcohol consumption, hepatitis B, hepatitis C), VCTE measurements, AST measures, FIB-4, and APRI. Participants were also restricted to adults aged ≥20 years.

**Figure 1.**
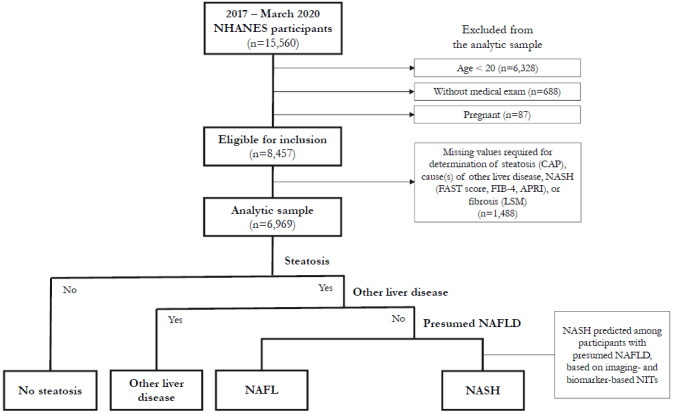
Study Population Flow Diagram Participants were classified as having presumed NAFLD based on presence of steatosis (liver fat content ≥5% based on CAP ≥302 dB/m) and lack of other liver disease (excessive alcohol consumption, hepatitis B, or hepatitis C). Among participants with presumed NAFLD, NASH was predicted based on FAST score, FIB-4, or APRI. Abbreviations: APRI, AST-to-Platelet Ratio Index; CAP, controlled attenuated parameter; FIB-4, Fibrosis-4 Index; LSM, liver stiffness measurement; NAFL, nonalcoholic fatty liver; NAFLD, nonalcoholic fatty liver disease; NASH, nonalcoholic steatohepatitis; NHANES, National Health and Nutrition Examination Survey; NIT, noninvasive test.

For participants included in the complete-case analysis, presumed NAFLD was identified using a multistepped approach similar to other NHANES studies^30,35,36^ **(Figure 1)**. First, participants were restricted to those with steatosis, then to those without common alternative causes of liver disease (ie, excessive alcohol consumption, hepatitis B, hepatitis C),^34^ as is common in other population-based studies.^25–27,36–42^ These participants were considered to have presumed NAFLD. NIT cutoffs for prediction of NASH were then applied among participants with presumed NAFLD. To assess the impact of different NITs on estimates of prevalence of NASH, 11 scenarios were conducted, varying the NIT and/or cutoff used for prediction of NASH.

For comparison, prevalence results estimated with the approach above were compared with estimates using screening algorithms recently proposed in clinical practice, including the Cirrhosis Prevention in NAFLD algorithm proposed by the AACE^18^ and the eligibility criteria for the Phase 3 MAESTRO-NASH clinical trial of resmetirom (NCT03900429).^43^ When the study was conducted, the AACE screening algorithm was the most contemporaneous statement from US societies (compared with a previous AASLD statement in 2018), and was the most explicit in NIT criteria for screening. Subsequently, the AASLD released a screening algorithm in 2023,^19^ broadly aligning with the AACE algorithm considered in this analysis. The eligibility criteria for MAESTRO-NASH were selected for comparison on the basis that the US FDA has granted Priority Review for resmetirom for treatment of adults with NASH with significant fibrosis, with a Prescription Drug User Fee Act date of March 14, 2024^44^; accordingly, the Phase 3 study eligibility criteria may be reflective of the population likely to be eligible for therapies approaching completion of clinical development.

### Identification of Presumed NAFLD

Identification of steatosis was defined as liver fat ≥5%,^15^ identified based on CAP data from VCTE. A cutoff of 302 dB/m was applied according to the value maximizing Youden’s index (balancing sensitivity and specificity) for prediction of liver fat ≥5%, as reported by Eddowes et al.^45^

Among participants identified with steatosis, liver disease was attributed to other causes before NAFLD, including excessive alcohol consumption, hepatitis B, and hepatitis C. Excessive alcohol consumption was defined as self-reported mean alcoholic drink consumption per day of >2 for men and >1 for women, in accordance with US Centers for Disease Control and Prevention guidance.^46^ Hepatitis B and hepatitis C were identified as self-report of ever being told by a healthcare professional that a participant had either condition.

### Prediction of NASH

Participants identified with presence of steatosis and lack of other liver disease were presumed to have NAFLD. Among individuals with presumed NAFLD, prediction of NASH was then applied using previously proposed cutoff values.^47^ For example, Newsome et al^20^ developed the FAST score for prediction of NAS ≥4 and fibrosis stage (F) ≥2 in a population with suspected NAFLD in England and validated the measure in 7 countries; rather than proposing a single cutoff value for prediction, they reported rule-out (0.35) and rule-in (0.67) values achieving 90% sensitivity and specificity, respectively. Subsequently, several studies have explored use of the FAST score and other NITs for prediction of NASH.^22,47–49^ For example, in a population with biopsy-proved NAFLD in South Korea, Lee et al^48^ compared performance of the FAST score to the NFS, FIB-4, and APRI for prediction of several definitions of NASH, reporting improved predictive performance of the FAST score over the biomarker-based NITs. In particular, for prediction of NAS ≥5, a FAST score ≥0.48 had sensitivity of 80.3% and specificity of 58.2% (C statistic = 0.752), and for prediction of NAS ≥4 and F ≥2, a FAST score ≥0.57 had sensitivity of 69.4% and specificity of 67.0% (C statistic = 0.714).

In this study, 11 scenario analyses were conducted using varying NITs and/or cutoffs for prediction of NASH, including 4 cutoff values for the FAST score, 4 cutoff values for FIB-4, and 3 cutoff values for APRI. For prediction with the FAST score, the rule-out (0.35) and rule-in (0.67) cutoff values reported by Newsome et al^20^ for at-risk NASH were used, as well as cutoff values balancing sensitivity and specificity for predicting NAS ≥5 (0.48) and NAS ≥4 and F ≥2 (0.57), as reported by Lee et al.^48^ For prediction with FIB-4, the rule-out (1.30) and rule-in (2.67) cutoff values reported by Shah et al^50^ for predicting “advanced fibrosis” (F3-F4) in NAFLD patients were used, as well as cutoff values balancing sensitivity and specificity for predicting NAS ≥5 (0.90) and NAS ≥4 and F ≥2 (1.59), as reported by Lee et al.^48^ For prediction with APRI, the rule-out (0.50) and rule-in (1.50) cutoff values reported by Wai et al^51^ for predicting “significant fibrosis” (F ≥3) in hepatitis C patients were used, as well as a cutoff value (0.70) reported by Lee et al^48^ to balance sensitivity and specificity for predicting NAS ≥5 or NAS ≥4 and F ≥2.

### Fibrosis Staging

For participants classified into the prevalent NASH population, the distribution across fibrosis stages was explored based on LSM from VCTE. VCTE measures LSM via a hand-held probe that captures the velocity of low-amplitude shear waves using ultrasound.^15^ Implementation of VCTE has been evaluated extensively, yielding reliable quality criteria to guide its use; however, ongoing limitations of VCTE in NAFLD/NASH are reported to include unclear optimal cutoff points for prediction of fibrosis stages, as well as limited diagnostic accuracy for earlier fibrosis stages.^15^ Indeed, proposed cutoff values for prediction of fibrosis stages vary in the literature. Boursier et al^47^ reported LSM (kPa) cutoff values of 4.6 for F1, 6.1 for F1/2, 8.8 for F2/3, 12.0 for F3, 18.0 for F3/4, and 38.6 for F4. Siddiqui et al^52^ reported cutoff values of 6.5 for ruling out advanced fibrosis and 12.1 for ruling out cirrhosis. Eddowes et al^45^ reported cutoff values maximizing sensitivity and specificity of 8.2 for F2, 9.7 for F3, and 13.6 for F4. Accordingly, rather than selecting specific cutoff values for classification of fibrosis stages, we summarized the distribution of LSM measurements for the prevalent NASH population for each scenario.

Estimates of the distribution of LSM should be interpreted in the context of the variation of LSM cutoffs. The evidence base suggests greater differences between cutoffs for fibrosis stages F3 and F4 compared with those for F2. For example, while Eddowes et al recommend a cutoff of 13.6 for F4, and Siddiqui et al. recommend a cutoff of 12.1 for ruling out cirrhosis (ie, 90% sensitivity for prediction of F4), Boursier et al^47^ report cutoffs of 12.0 for F3, 18.0 for F3/4, and 38.6 for F4. By contrast, there is closer agreement in cutoffs reported for F2. Boursier et al^47^ reported a cutoff of 8.8 for F2/3, and Eddowes et al^45^ reported a cutoff of 8.2 for F2. The AACE screening algorithm for Cirrhosis Prevention in NAFLD provides further context, indicating “low risk” for LSM <8 and “high risk” for LSM >12.

### Statistical Analysis

For the complete-case analysis population, means and standard errors of demographic and clinical characteristics were summarized overall and for the no-NAFLD vs presumed NAFLD groups, as reflected in **Table 1.** Variable definitions are provided in **Supplementary Table S1**. Statistical significance of differences between the no-NAFLD and presumed NAFLD groups was assessed using survey-weighted *t*-tests for continuous variables and survey-weighted χ^2^ tests with the Rao-Scott correction for categorical variables.

**Table 1.** Characteristics of the Overall Analytic Population, and No-NAFLD vs Presumed NAFLD Groups

	**Overall (N=6969)**	**No NAFLD (n=5127)**	**Presumed NAFLD (n=1842)**	***P* Value (Presumed NAFLD vs No NAFLD)**
	**Weighted % (SE)**			
Male sex (%)	49.4% (0.9%)	46.4% (0.9%)	58.1% (1.9%)	< .001
Age (years)				
Mean	48.2 (0.6)	47.2 (0.6)	50.9 (0.8)	< .001
20-39 (%)	35.6% (1.4%)	38.4% (1.5%)	27.5% (2.2%)	< .001
40-59 (%)	35.5% (1.0%)	34.2% (1.0%)	39.3% (2.3%)	
60-79 (%)	25.2% (1.4%)	23.5% (1.5%)	30.1% (2.0%)	
≥80 (%)	3.7% (0.3%)	3.9% (0.4%)	3.1% (0.4%)	
Race/ethnicity (%)				
Mexican American	8.5% (1.1%)	7.4% (1.0%)	11.6% (1.8%)	< .001
Other Hispanic	7.5% (0.8%)	7.5% (0.7%)	7.4% (1.1%)	
White	64.2% (2.4%)	64.1% (2.3%)	64.4% (3.4%)	
Black	10.4% (1.3%)	11.4% (1.4%)	7.6% (1.2%)	
Asian	5.3% (0.8%)	5.5% (0.9%)	4.7% (0.8%)	
Other (including mixed)	4.1% (0.4%)	4.0% (0.5%)	4.3% (0.7%)	
BMI category (%)				
Lean (<25)	26.3% (1.0%)	34.3% (1.2%)	3.4% (0.7%)	< .001
Overweight (25-29)	31.9% (0.8%)	35.7% (1.0%)	20.9% (1.7%)	
Obese (≥30)	41.8% (1.3%)	30.1% (1.2%)	75.8% (2.1%)	
Other liver disease (%)				
Excessive alcohol	7.9% (0.6%)	10.6% (0.8%)	0.0% (0.0%)	< .001
Hepatitis B	1.0% (0.2%)	1.3% (0.2%)	0.0% (0.0%)	< .001
Hepatitis C	1.7% (0.3%)	2.2% (0.4%)	0.0% (0.0%)	< .001
CV history (%)				
ASCVD	9.6% (0.7%)	8.5% (0.7%)	12.9% (1.3%)	< .001
HF	2.1% (0.3%)	1.6% (0.2%)	3.5% (0.8%)	.004
Any CVD (ASCVD and/or HF)	10.2% (0.7%)	9.0% (0.7%)	13.6% (1.4%)	< .001
CV risk factors				
Diabetes (%)	13.5% (0.5%)	8.9% (0.5%)	26.9% (1.6%)	< .001
Current cigarette smoking (%)	16.9% (1.1%)	18.6% (1.2%)	11.9% (1.1%)	< .001
Total cholesterol (mg/dL)				
Mean	187.4 (1.2)	186.9 (1.2)	188.7 (1.5)	.151
≥200 (%)	35.1% (1.2%)	34.8% (1.2%)	35.8% (2.1%)	.625
HDL-c (mg/dL)				
Mean	53.6 (0.4)	56.3 (0.4)	45.8 (0.6)	< .001
Low (%) (≤40 male, ≤50 female)	28.1% (1.1%)	22.0% (0.9%)	45.8% (2.4%)	< .001
SBP (mmHg)				
Mean	121.9 (0.4)	120.8 (0.5)	125.0 (0.6)	< .001
≥130 (%)	26.5% (1.1%)	24.5% (1.1%)	32.3% (2.3%)	< .001
≥140 (%)	14.0% (0.9%)	12.6% (0.8%)	17.8% (1.5%)	< .001
On medication for SBP (%)	24.1% (1.1%)	19.8% (0.9%)	36.5% (2.8%)	< .001

All counts are unweighted. NHANES analytic guidelines recommend sample size of ≥30 for reporting proportions, means, and variances.

In the base-case analysis, participants were classified as having presumed NAFLD based on presence of steatosis (liver fat content ≥5% based on CAP ≥302 dB/m) and lack of other liver disease (excessive alcohol consumption, hepatitis B, or hepatitis C).

Statistical significance of differences between the presumed NAFLD and no-NAFLD groups was assessed using survey-weighted t-tests for continuous variables and survey-weighted χ^2^ tests with the Rao-Scott correction for categorical variables.

Abbreviations: ASCVD, atherosclerotic cardiovascular disease; BMI, body mass index; CV, cardiovascular; CVD, cardiovascular disease; HDL-c, high-density lipoprotein cholesterol; HF, heart failure; NAFLD, nonalcoholic fatty liver disease; SBP, systolic blood pressure; SE, standard error.

Sampling weights provided by NCHS were used to estimate prevalence representative of the civilian, noninstitutionalized US population aged ≥20 years.^53^ In all analyses, recommended sampling weights and the Taylor series linearization variance approximation method were used to account for the complex survey design,^33^ and corresponding standard errors were calculated for all prevalence estimates. NHANES analytic guidelines recommend unweighted sample size ≥30 for reporting proportions, means, and variances.^53^ Population counts were calculated by multiplying prevalence estimates by the 2020 American Community Survey 1-year population estimates, which report a total of 256,824,592 persons aged ≥18 years.^54^

To compare the prevalence estimates based on prediction of NASH with NITs among participants with presumed NAFLD, scenario analyses were also conducted applying screening approaches recently proposed in clinical practice, including the AACE’s proposed Cirrhosis Prevention in NAFLD algorithm^18^ and the eligibility criteria for the MAESTRO-NASH clinical trial,^43^ the largest clinical study conducted in a biopsy-proven NASH population. The AACE algorithm recommends screening driven by presence of components of metabolic syndrome^55^ that predispose for NAFLD (including prediabetes, type 2 diabetes, obesity, ≥2 cardiometabolic risk factors, steatosis on imaging, or elevated aminotransferases), followed by fibrosis risk stratification first using FIB-4 (<1.30 = low, 1.30-2.67 = indeterminate, >2.67 = high) then with LSM by elastography (<8 kPa = low, 8-12 kPa = indeterminate, >12 kPa = high) if FIB-4 is indeterminate, and referral to a liver specialist for indeterminate-high risk. The MAESTRO-NASH eligibility criteria recommend screening based on medical history of NAFLD, presence of ≥3 risk factors for significant fibrosis (age >50 years, BMI >30 kg/m^2^, AST >20 U/L or AST/ALT ≥1, type 2 diabetes, dyslipidemia, hypertension, metabolic syndrome), LSM ≥8.5 kPa, and CAP ≥280 dB/m. Three scenarios were modeled for the MAESTRO-NASH eligibility criteria reflecting access to care, including no restriction on access to healthcare (scenario A), restricting to ≥1 healthcare visits in the last year (scenario B), and restricting to ≥1 healthcare visits in the last year and no evidence of other liver disease (scenario C) (see **Supplementary Material**).

All statistical analyses were conducted using R (version 4.0.2).

## RESULTS

### Study Population

Selection of the analytic sample is illustrated in **Figure 1**. The interview sample of the 2017-March 2020 NHANES survey cycle included 15,560 participants, of whom 8457 were eligible for the analysis. An additional 1488 participants were excluded due to missing data for identification of NASH, yielding an analytic sample of 6969 participants. NHANES analytic guidelines suggest consideration of imputation of missing values or use of adjusted sample weights if >10% of the data for an item are missing.^56^ Missing data were predominantly attributable to CAP, LSM, and AST measurements. Adjustments for missing VCTE data have not been used in other studies,^24,36^ and imputation was found not to significantly change results when conducted.^36^ Exploratory analysis was conducted in this study to assess the impact of reweighting the analytic sample for differences in age, sex, and race/ethnicity compared with all participants aged ≥20 years who completed the medical examination and was found to have minimal impact on results. Consequently, the original NHANES sample weights for the medical examination sample were used in this analysis.

In the analytic sample of 6969, 1842 participants were classified as having presumed NAFLD (**Table 1**). A higher proportion of individuals with vs without presumed NAFLD was male (58.1% vs 46.4%, *p* < .001) and a lower proportion was Black (7.6% vs 11.4%, *p* < .001). Individuals with vs without presumed NAFLD had a higher mean age (50.9 vs 47.2 years, *p* < .001). Prevalence of history of cardiovascular disease was higher with vs without presumed NAFLD (13.6% vs 9.0%, *p* < .001), as was the prevalence of several cardiovascular risk factors, including diagnosed diabetes (26.9% vs 8.9%, *p* < .001), systolic blood pressure (SBP) ≥130 mmHg (32.3% vs 24.5%, *p* < .001), SBP ≥140 mmHg (17.8% vs 12.6%, *p* < .001), use of medication for elevated SBP (36.5% vs 19.8%, *p* < .001), and low high-density lipoprotein cholesterol (HDL-C) level of ≤40 mg/dL for men or ≤50 mg/dL for women (45.8% vs 22.0%, *p* < .001). Levels of heavy metals (including arsenic, cadmium, lead, and mercury) and minerals (selenium) that may be associated with NASH pathophysiology generally fell within normal ranges.

### Prevalence of NASH

Prevalence of steatosis (CAP ≥302 dB/m) was estimated at 28.6%, among whom 89.6% did not report presence of other causes of liver disease (ie, excessive alcohol consumption, hepatitis B, and hepatitis C), yielding an estimated prevalence of presumed NAFLD of 25.6% (**Table 2**). Applying NITs for prediction of NASH in those with presumed NAFLD, prevalence of NASH was estimated to range from 1.3% to 4.8% based on FAST score cutoffs from 0.35 to 0.67, 0.4% to 12.3% based on FIB-4 cutoffs from 0.90 to 2.67, and 0.1% to 1.9% based on APRI cutoffs from 0.50 to 1.50. Scenario analysis applying the AACE screening algorithm^18^ suggested prevalence of 4.6%, and applying the eligibility criteria for the MAESTRO-NASH clinical trial^43^ yielded an estimate of 6.3%, which fell to 5.6% when restricting to participants with ≥1 healthcare visit in the past year, and to 5.0% when further restricting to those with no evidence of excessive alcohol consumption, hepatitis B, and hepatitis C.

**Table 2.** Estimated Prevalence of NASH Among US Adults

	**FAST**				**FIB-4**				**APRI**			**AACE Screening Pathway**	**MAESTRO-NASH Eligibility Criteria**		
	**≥0.35**	**≥0.48**	**≥0.57**	**≥0.67**	**≥0.90**	**≥1.30**	**≥1.59**	**≥2.67**	**≥0.50**	**≥0.70**	**≥1.50**		**Scenario A**	**Scenario B**	**Scenario C**
**Screening steps**															
Step 1	CAP ≥302 dB/m											“High-risk groups” for NAFLD	≥3 RFs for significant fibrosis		
													No medical history criterion	≥1 health visit in past year	≥1 visit, no other liver disease
Step 2	No other liver disease (hepatitis B/C, excessive alcohol consumption)											FIB-4 ≥1.3	CAP ≥280 dB/m		
Step 3	FAST ≥0.35	FAST ≥0.48	FAST ≥0.57	FAST ≥0.67	FIB-4 ≥0.90	FIB-4 ≥1.30	FIB-4 ≥1.59	FIB-4 ≥2.67	APRI ≥0.50	APRI ≥0.70	APRI ≥1.50	LSM ≥8 kPa *or* FIB-4 ≥2.67	LSM ≥8.5 kPa		
**Percentage of prior step meeting criterion**															
Step 1	28.6%											75.2%	49.9%	44.1%	39.9%
Step 2	89.6%											28.0%	61.1%	60.5%	60.5%
Step 3	18.6%	11.6%	8.1%	5.0%	47.9%	22.1%	12.6%	1.5%	7.5%	2.7%	0.3%	21.8%	20.7%	21.1%	20.8%
**Population estimate (in millions), by step**															
All adults	256.8														
Step 1	73.4											193.2	128.1	113.3	102.6
Step 2	65.7											54.1	78.3	68.5	62.1
Step 3	12.2	7.6	5.3	3.3	31.5	14.5	8.3	1.0	5.0	1.8	0.2	11.8	16.2	14.5	12.9
**Population prevalence estimate**															
Prevalence (%)	4.8	3.0	2.1	1.3	12.3	5.7	3.2	0.4	1.9	0.7	0.1	4.6	6.3%	5.6%	5.0%
SE (%)	0.4	0.2	0.2	0.2	0.8	0.5	0.3	0.1	0.2	0.1	0.0	0.5	(0.7%)	(0.6%)	(0.6%)
N	315	177	118	70	976	474	275	42	133	46	9	447	456	411	368

Prevalence estimates apply NHANES sampling weights, representative of the civilian, noninstitutionalized US population.

Adult population estimates are based on 256 824 592 persons aged ≥18 years, as reported in the 2020 American Community Survey (ACS) 1-year estimates.

NHANES analytic guidelines recommend sample size of ≥30 for reporting proportions, means, and variances.

Abbreviations: AACE, American Association of Clinical Endocrinology; APRI, AST-to-Platelet Ratio Index; CAP, controlled attenuation parameter; FAST, FibroScan + AST; FIB-4, Fibrosis-4 Index; LSM, liver stiffness measurement; NAFLD, nonalcoholic fatty liver disease; NASH, nonalcoholic steatohepatitis; RF, risk factor; SE, standard error.

Estimated population counts for the prevalent US adult NASH population are also presented in **Table 2**, comparing the NIT-based scenarios to those reflecting clinical screening algorithms. Analyses applying FAST-score cutoffs yielded prevalence estimates from 3.3 to 12.2 million, compared with estimates of 1.0 to 14.5 million and 0.2 to 5.0 million when applying FIB-4 and APRI cutoffs, respectively. In comparison, the AACE screening algorithm yielded estimated prevalence of 11.8 million, and the MAESTRO-NASH eligibility criteria a range from 12.9 million to 16.2 million.

### Fibrosis Stages Within NASH

**Figure 2.**
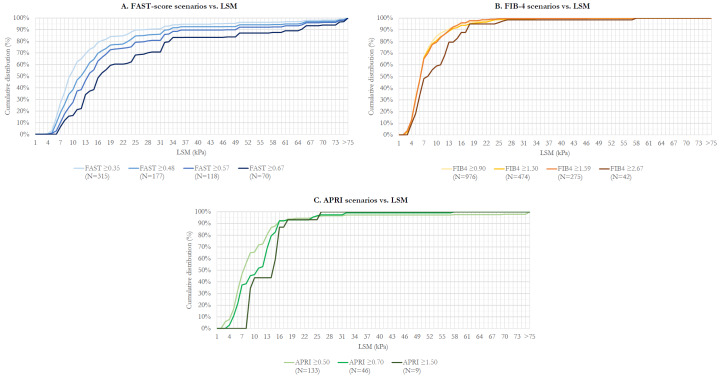
Cumulative Distribution Functions of NASH Prevalence vs LSM All counts are unweighted. NHANES analytic guidelines recommend sample size of ≥30 for reporting proportions, means, and variances. Abbreviations: APRI, AST-to-Platelet Ratio Index; FIB-4, Fibrosis-4 Index; LSM, liver stiffness measurement; NASH, nonalcoholic steatohepatitis.

Among prevalent NASH participants identified in the NIT-based analyses, fibrosis stages were explored based on distribution across the spectrum of LSM. The distribution of NASH prevalence is reflected by weighted cumulative distribution functions vs LSM (**Figure 2**). Difference in the cumulative distribution between LSM values gives the proportion of prevalent patients estimated to have LSM in a certain range. Although as described earlier, varying LSM cutoff values for fibrosis staging have been reported, for interpretation, cutoff values reported by Eddowes et al^45^ (8.2 for F2, 9.7 for F3, and 13.6 for F4) can be considered as an example. Underlying data for **Figure 2** are provided in **Supplementary Table S2**. Approximating based on Eddowes et al, with F2 as LSM ≥8 and <10, F3 as LSM ≥10 and <14, and F4 as LSM ≥14, **Figure 2** and **Table S2** reflect that among adults with NASH based on FAST ≥0.48, the distribution across F2, F3, and F4 is approximately 12%, 23%, and 38%, respectively.

The distribution of NASH patients skewed progressively toward higher fibrosis stages as FAST-score cutoff for prediction of NASH increased (**Figure 2**). By contrast, with use of FIB-4 for prediction of NASH, the distribution was relatively invariant for cutoffs of 0.90, 1.30, and 1.59, although skewed toward higher fibrosis stages using the 2.67 cutoff. For APRI, the distribution appeared to skew toward higher fibrosis stages when increasing the cutoff from 0.50 to 0.70 to 1.50; however, limited sample sizes using the 2 higher cutoffs (N=42 and N=9, respectively) limited reliability of these estimates.

## DISCUSSION

We estimated the nationally representative prevalence of NASH in US adults and assessed the impact of using imaging-based vs biomarker-based NITs on prevalence estimates. Using the FAST score, a combination of VCTE imaging and AST measurements, within participants with presumed NAFLD yielded prevalence estimates ranging from 1.3% to 4.8%, generally aligning with estimates from recent meta-analysis suggesting prevalence of biopsy-confirmed NASH from 1.5% to 6.5%.^6^ Use of biomarker-based NITs for prediction of NASH yielded a wider range of estimates from 0.4% to 12.3% with FIB-4, and a considerably lower range of estimates from 0.1% to 1.9% with APRI. Variation is to be expected between prevalence estimates, as the NITs and cutoff values used in this analysis were developed for prediction of advanced/ progressive liver disease with particular clinical features. APRI was developed for predicting “significant fibrosis” (F ≥3) in hepatitis C patients,^51^ and FIB-4 for predicting “advanced fibrosis” (F3-F4) in NAFLD patients.^50^ The FAST score was developed in suspected-NAFLD patients for prediction of at-risk NASH (ie, NAS ≥4 and F≥2), although the FAST score cutoffs applied in this analysis have been reported for rule-out and rule-in of at-risk NASH, as well as prediction of definite NASH (ie, NAS ≥5). Additionally, these NITs were developed in populations with clinical suspicion for liver disease/cirrhosis; accordingly, their predictive performance may vary when applied in an analysis of the general population, although our analysis did apply the NITs only among individuals with presumed NAFLD to address this. Despite these limitations of the use of NITs for estimating prevalence of NASH, our estimates using imaging-based NITs aligned relatively well with biopsy-based estimates from the literature, while the deviation using biomarker-based estimates suggests that additional information may be required for prediction of NASH. For example, the predictive performance of biomarker-based screening with FIB-4 may be improved by distinguishing cutoff values by age, as has been reported elsewhere.^57^In scenarios of this analysis applying screening algorithms from clinical practice, including the AACE screening algorithm^18^ and the eligibility criteria for the large Phase 3 MAESTRO-NASH clinical trial,^43^ estimated prevalence was found to be somewhat higher than scenarios predicting NASH based on imaging-based NIT cutoffs. In the imaging-based NIT scenarios, NASH was predicted first based on identification of presumed NAFLD via imaging for steatosis (ie, CAP measurements) and exclusion of other causes of liver disease, then on FAST score cutoff values. In clinical practice, current screening algorithms recommend initial screening based on broad metabolic risk factors and conditions associated with NAFLD/ NASH as well as steatosis measures if available, followed by use of biomarker- and/or imaging-based NITs as necessary. Initial screening based on imaging for steatosis may be infeasible due to the variable availability, complexity, and costs of large-scale VCTE measurement.^58,59^ For example, cost-effectiveness analyses of detection strategies for advanced fibrosis in NAFLD in a US setting^60^ suggest sequential application of biomarker-based (eg, FIB-4) and then imaging-based (eg, VCTE) NITs, aligning with current screening approaches.^18,19^ Nevertheless, application of current screening approaches also has challenges at the general-population level. In our analysis, >75% (193 million/257 million) of US adults were considered in high-risk groups for NAFLD screening based on the first step of the AACE algorithm (evidence of ≥1 of prediabetes or type 2 diabetes, obesity and/or ≥2 cardiometabolic risk factors, steatosis on imaging and/or elevated aminotransferases) (**Table 2**) and would therefore require fibrosis risk stratification with FIB-4. Of the 193 million who would require risk stratification with FIB-4, <10% had FAST score ≥0.35, suggesting limited positive predictive value of the first screening step, despite high sensitivity (the first screening step included >99% of those with FAST score ≥0.35). As previously reported, the limited positive predictive value of initial screening based on broad metabolic risk factors may contribute to lack of screening in primary care (eg, screening for NAFLD estimated at 46% in patients with obesity and diabetes in primary care^23^), with significant implications for management of NAFLD/NASH. For illustration, in our analysis, 13.5% of US adults (34.7 million) self reported being diagnosed with diabetes, among whom 96.4% (33.5 million) had ≥1 healthcare visit in the last year, indicating an opportunity for screening. Of these individuals, 14.1% (4.7 million) had a FAST score ≥0.35, suggesting that if screening were conducted for <50% of patients with diabetes in primary care, ≥2.5 million cases with FAST ≥0.35 could be missed. These findings underscore the importance of future research to improve the efficiency of screening processes for patients with more histologically advanced NAFLD/NASH and/or at risk of developing liver-related events, as others have recently explored.^61^

For validation, results of this analysis were compared with other NAFLD/NASH epidemiological findings from recent NHANES studies. For example, the scenario applying a FAST score cutoff of ≥0.67 was confirmed to yield prevalence (1.3%) aligning with that reported by Vilar-Gomez et al^62^ using NHANES 2017-2018 VCTE data. However, this study’s scenario applying a FAST score cutoff of ≥0.35 yielded lower prevalence than reported by Vilar-Gomez et al for the same cutoff (4.8% vs 5.8%). A key difference between the two studies’ approaches, beyond inclusion of additional data from 2019-2020 in our analysis, is that we restricted to participants with evidence of steatosis (CAP ≥302 dB/m) as a first step in identifying presumed NAFLD, while Vilar-Gomez et al did not. Restricting based on evidence of steatosis may help refine identification of NASH, particularly for individuals with FAST scores in the indeterminate range of 0.35 to 0.67 reported by Newsome et al.^20^ However, it might also exclude important cases of NASH as liver fat content has been observed to decline in later fibrosis stages and cirrhosis.

Potential limitations of the current analysis should be noted. First, NHANES consists of cross-sectional survey data from the non-institutionalized, civilian population in the US. Generalizability may be limited if applying the results to time points outside of the study period and to other populations and settings. For example, the prevalence of NASH may differ in incarcerated populations in the US (which do not contribute to these estimates), and, importantly, by settings of care. Additionally, while certain groups (low-income persons, older persons, and selected racial/ethnic populations) are oversampled in NHANES to allow for accurate population-level estimates, small-sample constraints may nonetheless challenge analyses of these subgroups. Second, certain variables in the analysis rely on self-reported data (eg, alcohol consumption, history of hepatitis B or C), which could result in misclassification. If these variables are underreported, this analysis may have included some participants with other liver diseases in the NASH estimates, which would result in overestimation of prevalence. Third, VCTE and AST measurements were missing for approximately 17.5% of participants eligible for inclusion in this analysis, which might bias results if not missing at random. However, previous analysis of the NHANES VCTE data suggests limited impact of imputing missing values,^36^ which we confirmed with exploratory analysis of adjustments to the NHANES sample weights. Fourth, VCTE is reported to have suboptimal performance for some patients, regardless of the probe size used (M or XL, both of which were used in the NHANES medical examinations). In particular, reduced accuracy has been reported in patients with BMI >30 kg/m^2^,^16,63^ and the majority of the presumed NAFLD group in this study had BMI in this range (**Table 1**). Additionally, inflammation and food intake may affect VCTE measurements,^16^ the potential for which cannot be excluded in the study population. Finally, in certain analyses (particularly applying APRI cutoff values of 0.70 and 1.50), small sample sizes may affect the reliability of estimates. Small sample sizes arose as NHANES has only collected VCTE measurements since the 2017-2018 survey cycle and the 2019-2020 cycle was suspended due to the COVID-19 pandemic. Additional research leveraging VCTE measurements collected in future cycles of NHANES may help address uncertainty in results of this analysis.

## CONCLUSION

With the anticipated emergence of novel interventions for treatment of NASH with moderate to significant fibrosis, the prevalence of treatment-eligible patients among US adults may be of particular interest to healthcare decision makers (eg, for planning screening and care delivery, projecting financial implications). In this study, the prevalence of NASH in US adults was estimated by applying measures commonly considered for screening (NITs), using the 2017-March 2020 NHANES survey cycles. Prevalence estimates ranged from 1.3% to 4.8% when using imaging-based NIT values (ie, FAST score), generally aligning with estimates in the literature of the prevalence of biopsy-confirmed NASH from 1.5% to 6.5%.^6^ Estimates using biomarker-based NIT values yielded a wider range from 0.4% to 12.3% with FIB-4, and a considerably lower range of estimates from 0.1% to 1.9% with APRI. Continuous NHANES survey cycles began collecting imaging-based NITs in 2017-2018; while this study used all available data from 2017-2020, analyses of data collected in future NHANES cycles will help address uncertainty around the prevalence of NASH estimated using NITs. Future research to clarify LSM cutoffs for prediction of liver fibrosis stages may further enhance understanding of the prevalence of treatment-eligible patients.

## Supplementary Material

Online Supplementary Material
