# Supplementary material for: Prevalence of Nonalcoholic Steatohepatitis and Associated Fibrosis Stages Among US Adults Using Imaging-Based vs Biomarker-Based Noninvasive Tests
Source: J Health Econ Outcomes Res. 2024 Feb 14;11(1):32–43. doi: 10.36469/001c.92223 (PMC10871169; doi:10.36469/001c.92223)
Supplement: Online Supplementary Material [file jheor_2024_11_1_92223_196336.pdf]

Journal of Health Economics and Outcomes Research

Online Supplementary Material

Prevalence of Nonalcoholic Steatohepatitis and Associated Fibrosis Stages Among US Adults Using Imaging-based vs Biomarker-based Noninvasive Tests. *JHEOR*. 2024;11(1):23-34. doi:10.36469/jheor.2024.92223

Table S1: Definitions of Variables in the Analysis **2**

Table S2: Distributions of NASH Prevalence vs LSM **5**

Scenario Definitions:

MAESTRO-NASH Eligibility Criteria **7**

AACE (2022) Screening Algorithm **8**

Checklist for Reporting of Survey Studies (CROSS) **9**

References **11**

This supplementary material has been provided by the authors to give readers additional information about their work.

This is an open-access article distributed under the terms of the Creative Commons Attribution 4.0 International License (CCBY-4.0). View this license's legal deed at http://creativecommons.org/licenses/by/4.0 and legal code at http://creativecommons.org/licenses/by/4.0/legalcode for more information.

Table S1. Definitions of Variables in the Analysis

Variable	Values/Levels	Calculation	Source Variables (NHANES)	Reference(s)
		**NONINVASIVE LIVER TESTS**		
APRI	Continuous	**Calculation:** (AST / upper limit of normal) / platelets<br>**Note**: AST ULN 37 male, 31 female<br>**Units**: AST in U/L, platelets n 10^9 cells/L	LBXSASSI, LBXPLTSI, RIAGENDR	Shah et al (2009)[1]<br>Nowicki and Pizzorno (2020)[2]
CAP	Continuous	**Calculation:** None, as reported (dB/m)	LUXCAPM	
FAST score	Continuous	**Calculation:**<br>exp[-1.65 + 1.07 x ln(LSM) + 2.66*10^{-8} x CAP^3 - 63.3 x AST^{-1}] /<br>[1 + exp(-1.65 + 1.07 x ln(LSM) + 2.66*10^{-8} * CAP^3 - 63.3 x AST^{-1})]<br>**Units**: CAP in dB/m, LSM in kPa	LUXCAPM, LUXSMED, LBXSASSI	Newsome et al (2020)[3]
FIB-4	Continuous	**Calculation:** (age x AST) / (platelet x √ALT)<br>**Units**: age in years, ALT in U/L, AST in U/L, platelets in 10^9 cells/L	AGEYR, LBXPLTSI, LBXSASSI, LBXSATSI	Shah et al (2009)[1]
LSM	Continuous	**Calculation:** None, as reported (kPa)	LUXSMED	
		**SAMPLE CHARACTERISTICS**		
Age	Continuous	**Calculation:** None, as reported (note: age ≥80 is coded as 80, due to censoring in NHANES)<br>**Units**: Years	RIDAGEYR	
BMI category	Lean, overweight, obese	**Calculation:** Lean if BMI <25, overweight if BMI ≥25 and <30, obese if BMI ≥30	BMXBMI	CDC (2022)[4]
CV history (%)				
ASCVD	Indicator (0/1)	**Calculation:**<br>self-reported history of coronary heart disease, heart attack, or stroke<br>*or*<br>self-reported history of angina pectoris *or* angina pectoris according to the Rose questionnaire	MCQ160C, MCQ160D, MCQ160E, MCQ160F, CDQ001-006, CDQ009D, CDQ009E, CDQ009F, CDQ009G	See NHANES CDQ documentation for definition of angina pectoris according to the Rose questionnaire
HF	Indicator (0/1)	**Calculation:** self-reported history of heart failure	MCQ160B	
Any CVD	Indicator (0/1)	**Calculation:** ASCVD and/or HF	Derived from ASCVD and HF	
CV risk factors				
Current cigarette smoking	Indicator (0/1)	**Calculation:** self-report of ever having smoked ≥100 cigarettes, and currently smoking every day or some days	SMQ020, SMQ040	
Diabetes	Indicator (0/1)	**Calculation:** self-report of a participant ever having been told they had diabetes or borderline diabetes by a health professional	DIQ010	

Table S1. Definitions of Variables in the Analysis

Variable	Values/Levels	Calculation	Source Variables (NHANES)	Reference(s)
Total cholesterol				
mg/dL	Continuous	**Calculation:** None, as reported	LBXTC	
High total cholesterol	Indicator (0/1)	**Calculation:** Total cholesterol ≥200 mg/dL	Derived from total cholesterol	
HDL-C				
mg/dL	Continuous	**Calculation:** None, as reported	LBDHDD	
Low HDL-C	Indicator (0/1)	**Calculation:** male ≤40 mg/dL, female ≤50 mg/dL	Derived from HDL-C	AHA (2021)[5]
Systolic blood pressure				
mmHg	Continuous	**Calculation:** Mean of up to 3 measures reported	BPXOSY1-3	
SBP ≥130 mmHg	Indicator (0/1)	**Calculation:** Mean SBP ≥130 mmHg	Derived from SBP	AHA (2021)[5]
SBP ≥140 mmHg	Indicator (0/1)	**Calculation:** Mean SBP ≥140 mmHg		
On medication for SBP	Indicator (0/1)	**Calculation:** self-report of a participant ever having been told they had hypertension, prescribed medication for hypertension, and currently taking the medication	BPQ020, BPQ040A, BPQ050A	
Heavy-metal exposure				
High arsenic	Indicator (0/1)	**Calculation:** arsenic (urine) ≥35 ug/L	URXUAS	Keil et al (2011)[6]
High cadmium	Indicator (0/1)	**Calculation:** cadmium (blood) ≥5 ug/L	LBXBCD	
High lead	Indicator (0/1)	**Calculation:** lead (blood) ≥30 ug/L	LBXBPB	
High mercury	Indicator (0/1)	**Calculation:** mercury (blood) ≥15 ug/L	LBXTHG	
Low selenium	Indicator (0/1)	**Calculation:** selenium <120 ug/L	LBXBSE	Wimmer et al (2014)[7]
High selenium	Indicator (0/1)	**Calculation:** selenium >160 ug/L	LBXBSE	
Other liver disease	Indicator (0/1)	**Calculation:** Reflects evidence/history of excessive alcohol consumption, hepatitis B, or hepatitis C. Excessive alcohol consumption was defined as mean drinks per day >2 for men and >1 for women (aligning with the CDC), among participants who had ever consumed alcohol (ALQ111) and reported drinking alcohol in the past 12 months (ALQ121). Hepatitis B and C were defined by self-report of a participant ever having been told they had either condition by a health professional.	ALQ111, ALQ121, ALQ130, HEQ010, HEQ030	CDC (2019)[8]

Table S1. Definitions of Variables in the Analysis

Variable	Values/Levels	Calculation	Source Variables (NHANES)	Reference(s)
Race/ethnicity	Asian, Black, Mexican American, Other Hispanic, White, Other (incl. mixed)	**Calculation:**<br>Mexican if RIDRETH3=1, Other Hispanic if RIDRETH3=2, White if RIDRETH3=3, Black if RIDRETH3=4, Asian if RIDRETH3=6, Other if RIDRETH3=7	RIDRETH3	
Sex	Female, male	**Calculation:** male if RIAGENDR=1, female if RIAGENDR=2, NA otherwise	RIAGENDR	

Abbreviations: ALT, alanine transaminase; APRI, AST-to-Platelet Ratio Index; ASCVD, atherosclerotic cardiovascular disease; AST, aspartate transaminase; BMI, body mass index; CAP, controlled attenuation parameter; CDC, Centers for Disease Control and Prevention; CV, cardiovascular; CVD, cardiovascular disease; FAST, FibroScan + AST; FIB-4, Fibrosis-4 index; HDL-c, high-density lipoprotein cholesterol; HF, heart failure; LSM, liver stiffness measurement; SBP, systolic blood pressure; ULN, upper limit of normal.

Table S2. Distributions of NASH Prevalence vs LSM

LSM	FAST ≥0.35 (n=315)	FAST ≥0.48 (n=177)	FAST ≥0.57 (n=118)	FAST ≥0.67 (n=70)	FIB-4 ≥0.90 (n=976)	FIB-4 ≥1.30 (n=474)	FIB-4 ≥1.59 (n=275)	FIB-4 ≥2.67 (n=42)	APRI ≥0.50 (n=133)	APRI ≥0.70 (n=46)	APRI ≥1.50 (n=9)
0-1	–	–	–	–	–	–	–	–	–	–	–
1-2	–	–	–	–	–	–	–	–	–	–	–
2-3	–	–	–	–	1.8%	2.8%	3.8%	–	5.9%	–	–
3-4	0.6%	0.3%	–	–	9.0%	9.8%	8.9%	9.5%	1.9%	3.0%	–
4-5	2.7%	0.8%	0.9%	–	20.5%	19.3%	17.8%	8.9%	9.0%	7.7%	–
5-6	11.2%	8.6%	2.0%	–	16.6%	15.6%	17.0%	15.9%	16.3%	10.9%	–
6-7	12.3%	8.8%	7.1%	6.3%	18.0%	18.8%	17.6%	14.0%	13.6%	15.7%	–
7-8	9.2%	7.3%	7.4%	5.6%	8.3%	6.0%	5.1%	2.2%	9.5%	1.1%	–
8-9	12.5%	8.5%	5.5%	3.7%	5.8%	5.8%	7.1%	5.3%	8.7%	6.8%	34.5%
9-10	6.4%	4.0%	4.4%	0.5%	4.5%	2.6%	1.9%	3.2%	0.7%	1.0%	8.9%
10-11	7.5%	8.5%	9.8%	5.1%	2.7%	3.6%	4.2%	1.1%	6.1%	5.5%	–
11-12	2.5%	3.5%	1.2%	0.8%	1.8%	2.0%	2.9%	8.4%	0.8%	1.3%	–
12-13	4.4%	5.4%	7.7%	12.0%	1.7%	2.6%	3.1%	11.0%	8.0%	15.6%	–
13-14	3.8%	5.9%	6.4%	3.0%	1.8%	2.3%	3.0%	–	6.1%	10.6%	–
14-15	1.7%	2.8%	2.8%	1.3%	1.6%	0.7%	1.3%	3.1%	1.5%	3.4%	15.8%
15-16	4.4%	5.3%	7.6%	10.0%	1.5%	2.0%	2.3%	5.2%	4.0%	9.7%	27.6%
16-17	1.4%	2.2%	3.2%	4.4%	0.4%	–	–	–	–	–	–
17-18	1.3%	2.0%	2.9%	2.9%	0.6%	1.1%	1.8%	7.3%	1.9%	0.7%	6.4%
18-19	2.2%	2.7%	3.6%	3.7%	0.4%	0.7%	–	–	–	–	–
19-20	0.3%	0.4%	0.6%	1.0%	0.1%	0.2%	–	–	0.7%	–	–
20-21	0.1%	0.2%	0.3%	–	0.2%	0.4%	0.7%	–	–	–	–
21-22	0.2%	0.3%	0.4%	–	0.1%	0.2%	0.1%	–	–	–	–
22-23	1.2%	1.9%	0.4%	0.7%	0.7%	1.0%	0.4%	–	–	–	–
23-24	2.1%	2.2%	0.8%	1.3%	0.8%	0.9%	–	–	0.8%	2.4%	–
24-25	1.8%	2.9%	3.9%	5.9%	0.3%	0.2%	0.4%	1.1%	0.8%	1.2%	–
25-26	0.1%	0.2%	0.2%	0.4%	0.0%	0.1%	0.2%	1.3%	0.3%	0.7%	6.8%
26-27	0.1%	0.1%	0.2%	0.3%	0.0%	0.1%	0.1%	1.0%	0.2%	–	–
27-28	0.3%	0.5%	0.7%	1.2%	0.1%	–	–	–	–	–	–
28-29	0.2%	0.3%	0.4%	0.7%	–	–	–	–	–	–	–
29-30	0.1%	0.1%	–	–	–	–	–	–	–	–	–
30-31	0.1%	0.2%	–	–	–	–	–	–	–	–	–
31-32	2.2%	3.6%	5.1%	8.3%	–	–	–	–	0.6%	1.8%	–
32-33	0.1%	0.2%	0.3%	0.6%	0.1%	–	–	–	–	–	–
33-34	1.1%	1.7%	2.3%	3.7%	0.4%	0.8%	–	–	–	–	–
34-35	–	–	–	–	–	–	–	–	–	–	–
35-36	0.5%	0.9%	1.2%	–	–	–	–	–	–	–	–
36-37	0.0%	0.1%	0.1%	0.1%	0.0%	–	–	–	–	–	–
37-38	–	–	–	–	–	–	–	–	–	–	–
38-39	–	–	–	–	–	–	–	–	–	–	–
39-40	0.1%	–	–	–	–	–	–	–	–	–	–
40-41	–	–	–	–	–	–	–	–	–	–	–
41-42	–	–	–	–	–	–	–	–	–	–	–
42-43	–	–	–	–	–	–	–	–	–	–	–
43-44	0.4%	–	–	–	–	–	–	–	–	–	–

Table S2. Distributions of NASH Prevalence vs LSM

LSM	FAST ≥0.35 (n=315)	FAST ≥0.48 (n=177)	FAST ≥0.57 (n=118)	FAST ≥0.67 (n=70)	FIB-4 ≥0.90 (n=976)	FIB-4 ≥1.30 (n=474)	FIB-4 ≥1.59 (n=275)	FIB-4 ≥2.67 (n=42)	APRI ≥0.50 (n=133)	APRI ≥0.70 (n=46)	APRI ≥1.50 (n=9)
44-45	–	–	–	–	–	–	–	–	–	–	–
45-46	–	–	–	–	–	–	–	–	–	–	–
46-47	0.3%	0.1%	0.2%	0.3%	0.1%	0.1%	0.1%	–	–	–	–
47-48	–	–	–	–	–	–	–	–	–	–	–
48-49	–	–	–	–	–	–	–	–	–	–	–
49-50	0.9%	1.5%	2.1%	3.4%	–	–	–	–	–	–	–
50-51	–	–	–	–	–	–	–	–	–	–	–
51-52	–	–	–	–	–	–	–	–	–	–	–
52-53	–	–	–	–	–	–	–	–	–	–	–
53-54	–	–	–	–	–	–	–	–	–	–	–
54-55	–	–	–	–	–	–	–	–	–	–	–
55-56	–	–	–	–	–	–	–	–	–	–	–
56-57	–	–	–	–	–	–	–	–	–	–	–
57-58	0.1%	0.2%	0.3%	0.5%	0.0%	0.1%	0.2%	1.5%	0.3%	0.9%	–
58-59	–	–	–	–	–	–	–	–	–	–	–
59-60	–	–	–	–	–	–	–	–	–	–	–
60-61	0.4%	0.6%	0.9%	1.4%	–	–	–	–	–	–	–
61-62	–	–	–	–	–	–	–	–	–	–	–
62-63	–	–	–	–	–	–	–	–	–	–	–
63-64	–	–	–	–	–	–	–	–	–	–	–
64-65	0.4%	0.6%	0.9%	1.4%	–	–	–	–	–	–	–
65-66	0.8%	1.3%	1.8%	2.9%	–	–	–	–	–	–	–
66-67	–	–	–	–	–	–	–	–	–	–	–
67-68	–	–	–	–	–	–	–	–	–	–	–
68-69	–	–	–	–	–	–	–	–	–	–	–
69-70	0.2%	0.3%	0.3%	0.5%	0.1%	–	–	–	0.3%	–	–
70-71	–	–	–	–	–	–	–	–	–	–	–
71-72	–	–	–	–	–	–	–	–	–	–	–
72-73	–	–	–	–	–	–	–	–	–	–	–
73-74	0.7%	1.1%	1.6%	2.6%	–	–	–	–	–	–	–
74-75	0.1%	0.1%	0.2%	0.3%	–	–	–	–	–	–	–
≥75	1.0%	1.5%	1.9%	3.1%	0.0%	0.1%	–	–	2.1%	–	–

SCENARIO ANALYSIS DEFINITIONS

MAESTRO-NASH eligibility criteria

Scenarios based on the MAESTRO-NASH eligibility criteria were informed by the algorithm depicted in Loomba et al. (2022),[9] as replicated below.

In the NHANES 2017-March 2020 cycle, MRI-PDFF and liver biopsy were not conducted. Consequently, the following screening steps were modeled:

Step 1: Evidence of ≥3 risk factors of significant fibrosis, including:

- Age >50 years
- BMI >30 kg/m2
- AST >20 U/L or AST/ALT ≥1
- Diabetes (self-report of being told by a healthcare professional that one had diabetes or “borderline” diabetes)
- Dyslipidemia (total cholesterol ≥200 mg/dL, triglycerides ≥150 mg/dL, LDL-C ≥130 mg/dL, or low HDL-C defined as <50 mg/dL for women and <40 mg/dL for men)
- Hypertension (systolic blood pressure ≥130 mmHg or diastolic blood pressure ≥85 mmHg)
- Metabolic syndrome (≥3 of: HbA1c ≥5.7% and/or treatment for high blood glucose, waist circumference >35 inches for women and >40 inches for men, hypertension and/or treatment for high blood pressure, triglycerides >150 mg/dL or treatment for high cholesterol, low HDL-C)

Step 2: Steatosis reflected by controlled attenuation parameter (CAP) ≥280 dB/m, from VCTE

Step 3: Liver stiffness measure (LSM) ≥8.5 kPa, from VCTE

In addition to the steps above, three scenarios were modeled varying access to care, reflective of individuals who might be diagnosed with NASH in practice. These included:

- **Scenario A**: No restriction on access to care
- **Scenario B:** Initial restriction to individuals with ≥1 healthcare visits in last year (NHANES variable HUQ051)
- **Scenario C:** Initial restriction to individuals with ≥1 healthcare visits in last year (NHANES variable HUQ051) and no evidence of other liver disease (excessive alcohol consumption, hepatitis B, or hepatitis C)

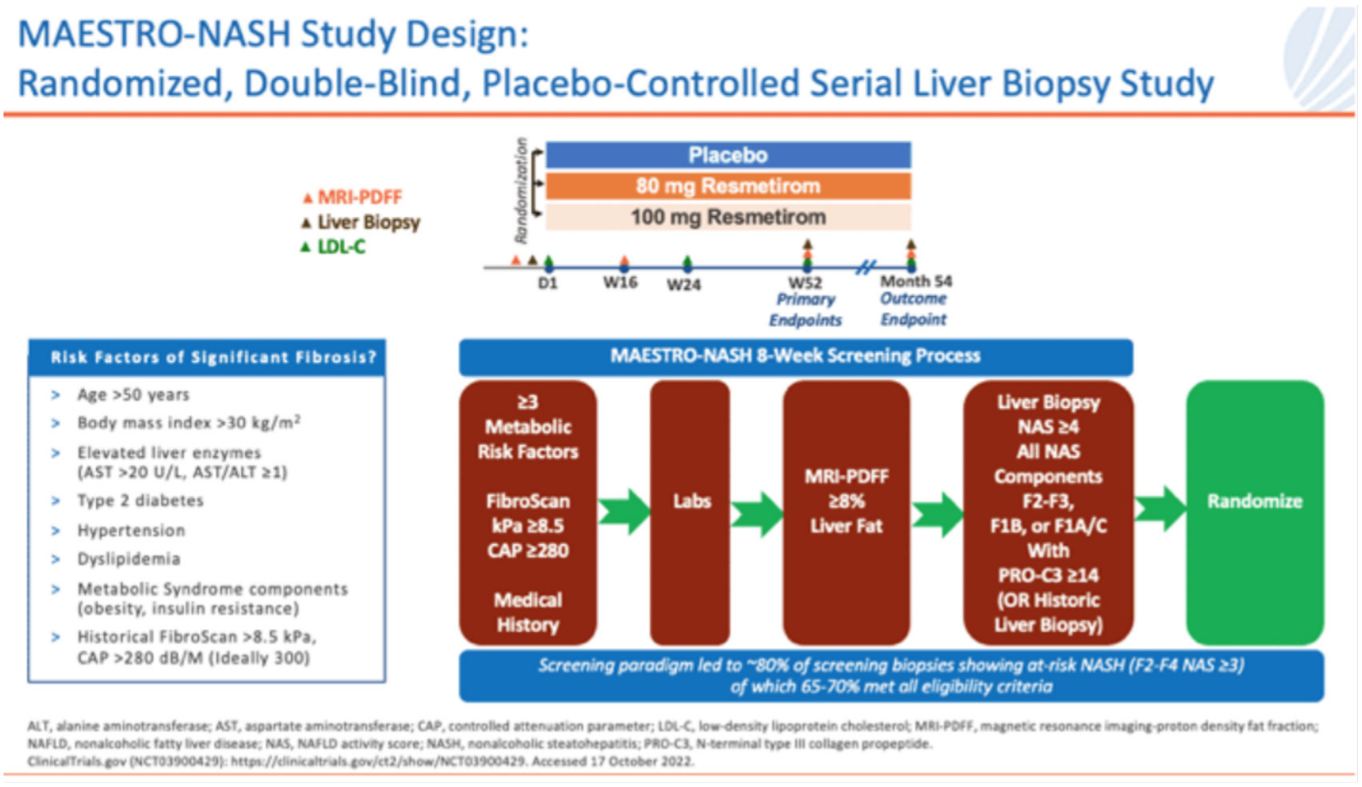

Source: Replicated from Loomba et al (2022).[9]

AACE (2022) Screening Algorithm

The scenario based on the AACE (2022) "Cirrhosis Prevention in NAFLD" screening algorithm was informed by the process described in Cusi et al (2022),[10] as replicated below.

In the NHANES 2017-March 2020 cycle, the ELF blood test was not conducted. Consequently, the following screening steps were modeled:

Step 1: Evidence of ≥1 of:

- Prediabetes or diabetes (self-report of being told by a healthcare professional that one had diabetes, "borderline" diabetes, or prediabetes)
- Obesity (BMI >30 kg/m2) and/or ≥2 cardiometabolic risk factors (HbA1c ≥5.7% and/or treatment for high blood glucose, waist circumference >35 inches for women and >40 inches for men, hypertension and/or treatment for high blood pressure, triglycerides >150 mg/dL or treatment for high cholesterol, HDL-C <50 mg/dL for women and <40 mg/dL for men)
- Steatosis on imaging (CAP ≥288 dB/m) and/or elevated aminotransferases (ALT >30 U/L or AST >30 U/L)

Step 2: FIB-4 ≥1.30

Step 3: FIB-4 >2.67 or LSM ≥8.0 kPa

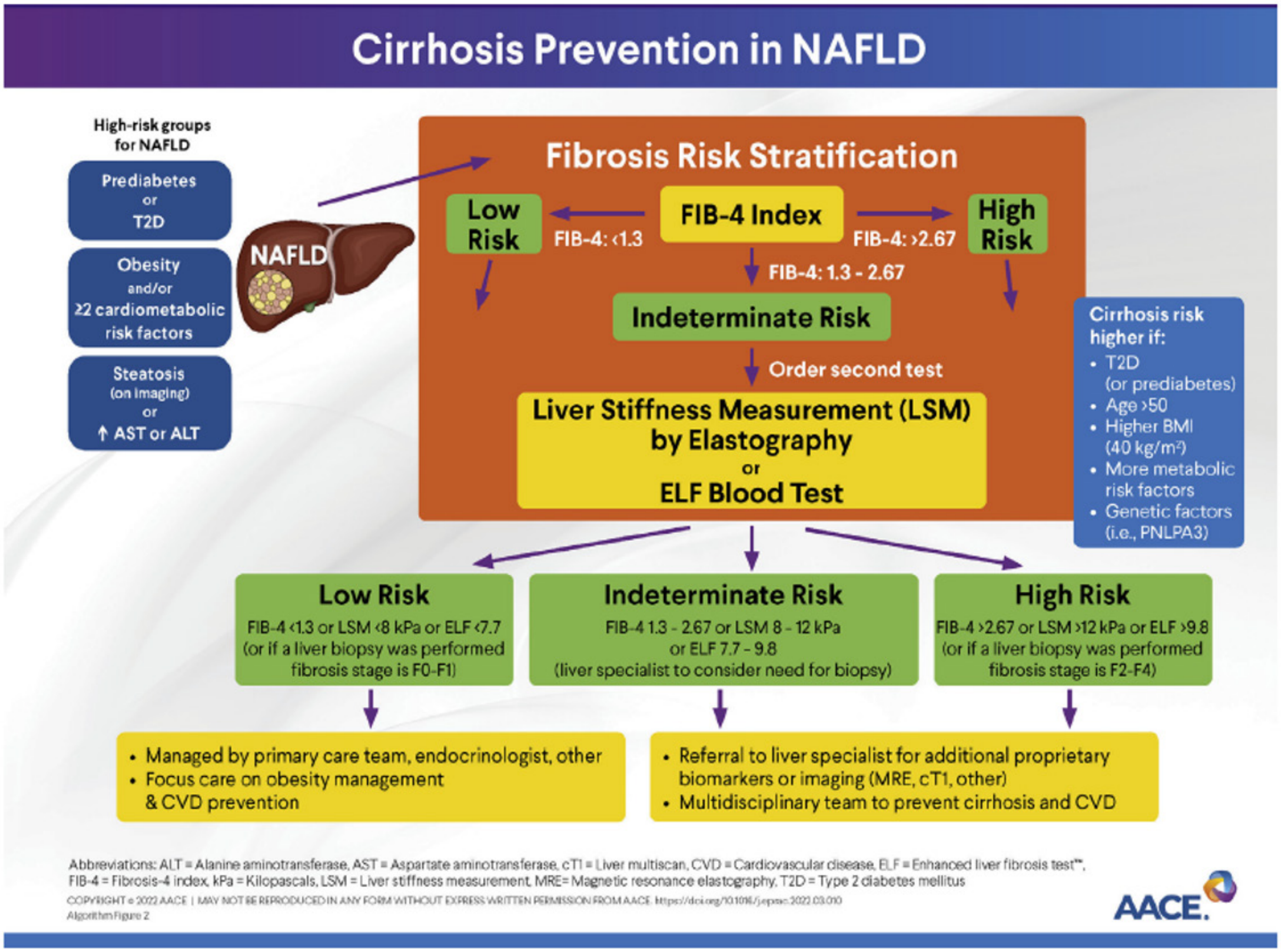

Source: Replicated from Cusi et al (2022).[10]

CHECKLIST FOR REPORTING OF SURVEY STUDIES (CROSS)

Section/Topic	Item	Item Description	Reported on Page No.
**Title and abstract**			
Title and abstract	1a	State the word "survey" along with a commonly used term in title or abstract to introduce the study's design.	32
	1b	Provide an informative summary in the abstract, covering background, objectives, methods, findings/results, interpretation/discussion, and conclusions.	32
**Introduction**			
Background	2	Provide a background about the rationale of study, what has been previously done, and why this survey is needed.	32-33
Purpose/aim	3	Identify specific purposes, aims, goals, or objectives of the study.	33
**Methods**			
Study design	4	Specify the study design in the methods section with a commonly used term (eg, cross-sectional or longitudinal).	33
	5a	Describe the questionnaire (eg, number of sections, number of questions, number and names of instruments used).	NHANES documentation
Data collection methods	5b	Describe all questionnaire instruments that were used in the survey to measure particular concepts. Report target population, reported validity and reliability information, scoring/classification procedure, and reference links (if any).	NHANES documentation
	5c	Provide information on pretesting of the questionnaire, if performed (in the article or in an online supplement). Report the method of pretesting, number of times questionnaire was pre-tested, number and demographics of participants used for pretesting, and the level of similarity of demographics between pre-testing participants and sample population.	NHANES documentation
	5d	Questionnaire if possible, should be fully provided (in the article, or as appendices or as an online supplement).	NHANES documentation
Sample characteristics	6a	Describe the study population (ie, background, locations, eligibility criteria for participant inclusion in survey, exclusion criteria).	NHANES documentation
	6b	Describe the sampling techniques used (eg, single stage or multistage sampling, simple random sampling, stratified sampling, cluster sampling, convenience sampling). Specify the locations of sample participants whenever clustered sampling was applied.	NHANES documentation
	6c	Provide information on sample size, along with details of sample size calculation.	35, 36, and Figure 1
	6d	Describe how representative the sample is of the study population (or target population if possible), particularly for population-based surveys.	35, 37
Survey administration	7a	Provide information on modes of questionnaire administration, including the type and number of contacts, the location where the survey was conducted (eg, outpatient room or by use of online tools, such as SurveyMonkey).	NHANES documentation
	7b	Provide information of survey's time frame, such as periods of recruitment, exposure, and follow-up days.	NHANES documentation
	7c	Provide information on the entry process:<br>–>For non-web-based surveys, provide approaches to minimize human error in data entry.<br>–>For web-based surveys, provide approaches to prevent "multiple participation" of participants.	NHANES documentation
Study preparation	8	Describe any preparation process before conducting the survey (eg, interviewers' training process, advertising the survey).	NHANES documentation
Ethical considerations	9a	Provide information on ethical approval for the survey if obtained, including informed consent, institutional review board [IRB] approval, Helsinki declaration, and good clinical practice [GCP] declaration (as appropriate).	34
	9b	Provide information about survey anonymity and confidentiality and describe what mechanisms were used to protect unauthorized access.	NHANES documentation
Statistical analysis	10a	Describe statistical methods and analytical approach. Report the statistical software that was used for data analysis.	34, 35, and Figure 1

Section/Topic	Item	Item Description	Reported on Page No.
	10b	Report any modification of variables used in the analysis, along with reference (if available).	Table S1
	10c	Report details about how missing data was handled. Include rate of missing items, missing data mechanism (ie, missing completely at random [MCAR], missing at random [MAR] or missing not at random [MNAR]) and methods used to deal with missing data (eg, multiple imputation).	35, 37, 40
	10d	State how non-response error was addressed.	34-35, 37, 40
	10e	For longitudinal surveys, state how loss to follow-up was addressed.	N/A
	10f	Indicate whether any methods such as weighting of items or propensity scores have been used to adjust for non-representativeness of the sample.	35, 37
	10g	Describe any sensitivity analysis conducted.	34-35 and Table 2
**Results**			
Respondent characteristics	11a	Report numbers of individuals at each stage of the study. Consider using a flow diagram, if possible.	Figure 1
	11b	Provide reasons for non-participation at each stage, if possible.	34-35 and Figure 1
	11c	Report response rate, present the definition of response rate or the formula used to calculate response rate.	NHANES documentation
	11d	Provide information to define how unique visitors are determined. Report number of unique visitors along with relevant proportions (eg, view proportion, participation proportion, completion proportion).	N/A
Descriptive results	12	Provide characteristics of study participants, as well as information on potential confounders and assessed outcomes.	35, 37, and Table 1
Main findings	13a	Give unadjusted estimates and, if applicable, confounder-adjusted estimates along with 95% confidence intervals and *p* values.	Tables 1-2
	13b	For multivariable analysis, provide information on the model building process, model fit statistics, and model assumptions (as appropriate).	N/A
	13c	Provide details about any sensitivity analysis performed. If there are considerable amount of missing data, report sensitivity analyses comparing the results of complete cases with that of the imputed dataset (if possible).	35, 37, 40, and Table 2
**Discussion**			
Limitations	14	Discuss the limitations of the study, considering sources of potential biases and imprecisions, such as non-representativeness of sample, study design, important uncontrolled confounders.	40
Interpretations	15	Give a cautious overall interpretation of results, based on potential biases and imprecisions and suggest areas for future research.	37, 40
Generalizability	16	Discuss the external validity of the results.	37, 40
**Other sections**			
Role of funding source	17	State whether any funding organization has had any roles in the survey's design, implementation, and analysis.	Reported online
Conflict of interest	18	Declare any potential conflict of interest.	Reported online
Acknowledgments	19	Provide names of organizations/persons that are acknowledged along with their contribution to the research.	Reported online

Source: Sharma et al (2021).[11]

REFERENCES

1. Shah AG, Lydecker A, Murray K, Tetri BN, Contos MJ, Sanyal AJ. Comparison of noninvasive markers of fibrosis in patients with nonalcoholic fatty liver disease. *Clin Gastroenterol Hepatol*. 2009;7(10):1104-12. doi:10.1016/j.cgh.2009.05.033
2. Nowicki J, Pizzorno JE. Nonalcoholic fatty liver disease and nonalcoholic steatohepatitis. In: Pizzorno JE, Murray MT, eds. *Textbook of Natural Medicine (Fifth Edition)*. Churchill Livingstone; 2020:1600-1604.e3.
3. Newsome PN, Sasso M, Deeks JJ, et al. FibroScan-AST (FAST) score for the non-invasive identification of patients with non-alcoholic steatohepatitis with significant activity and fibrosis: a prospective derivation and global validation study. *Lancet Gastroenterol Hepatol*. 2020;5(4):362-373. doi:10.1016/S2468-1253(19)30383-8
4. Centers for Disease Control and Prevention. Adult obesity facts. March 17, 2022. Accessed October 8, 2023. https://www.cdc.gov/obesity/data/adult.html.
5. American Heart Association. What is metabolic syndrome? 2021. Accessed October 8, 2023. https://www.heart.org/-/media/Files/Health-Topics/Answers-by-Heart/What-Is-Metabolic-Syndrome.pdf.
6. Keil DE, Berger-Ritchie J, McMillin GA. Testing for Toxic Elements: A focus on arsenic, cadmium, lead, and mercury. *Lab Med*. 2011;42(12):735-742. doi:10.1309/LMYKGU05BEPE7IAW
7. Wimmer I, Hartmann T, Brustbauer R, Minear G, Dam K. Selenium levels in patients with autoimmune thyroiditis and controls in lower Austria. *Horm Metab Res*. Sep 2014;46(10):707-9. doi:10.1055/s-0034-1377029
8. Centers for Disease Control and Prevention. Alcohol and public health. December 30, 2019. Accessed October 8, 2023. https://www.cdc.gov/alcohol/onlinemedia/infographics/excessive-alcohol-use.html.
9. Loomba R, Harrison SA, Taub RA, et al. Utility of FIB-4, MRE, MRI-PDFF, & FibroScan to identify patients with at-risk F2-F3 NASH based on screening data from a 2000 patient biopsy-confirmed cohort of resmetirom phase 3 clinical trial (MAESTRO-NASH). American Association for the Study of Liver Disease. The Liver Meeting, Washington DC, November 4-8 2022. Accessed January 20, 2024. https://www.natap.org/2022/AASLD/AASLD_13.htm.
10. Cusi K, Isaacs S, Barb D, et al. American Association of Clinical Endocrinology Clinical Practice Guideline for the Diagnosis and Management of Nonalcoholic Fatty Liver Disease in Primary Care and Endocrinology Clinical Settings: Co-Sponsored by the American Association for the Study of Liver Diseases (AASLD). *Endocr Pract*. 2022;28(5):528-562. doi:10.1016/j.eprac.2022.03.010
11. Sharma A, Minh Duc NT, Luu Lam Thang T, et al. A Consensus-Based Checklist for Reporting of Survey Studies (CROSS). *J Gen Intern Med*. 2021;36(10):3179-3187. doi:10.1007/s11606-021-06737-1
